# Design of high performance p-type sensitizers with pyridinium derivatives as the acceptor by theoretical calculations†

**DOI:** 10.1039/d0ra00610f

**Published:** 2020-03-12

**Authors:** Zhi-Dan Sun, Jiang-Shan Zhao, Karuppasamy Ayyanar, Xue-Hai Ju, Qi-Ying Xia

**Affiliations:** Key Laboratory of Soft Chemistry and Functional Materials of MOE, School of Chemical Engineering, Nanjing University of Science and Technology Nanjing 210094 P. R. China xhju@njust.edu.cn; School of Chemistry and Chemical Engineering, Linyi University Linyi P. R. China xiaqiying@163.com

## Abstract

Based on triphenylamine as an electron donor and thiophene as a π-linker, Series P and A p-type sensitizers were designed to investigate the effects of the different acceptors on the properties of the sensitizers. The optimized molecular structures, electronic and optical properties were investigated by density functional theory (DFT) and time-dependent DFT (TD-DFT). The results showed that the properties of the dyes can be tuned by the introduction of the different electron-withdrawing groups to the N atom in the pyridinium acceptor. Compared with the synthesized Series P dyes used in p-type sensitizers, the properties of Series A dyes, except for two dyes that cannot be used as p-type sensitizers, are improved by means of modifying pyridinium acceptors. Due to the suitable electron-withdrawing ability of the hexafluorodiacetylamino group in its acceptor, A6 has the narrowest energy gap (1.90 eV), the largest driving force of hole injection (Δ*G*_inj_, −0.68 eV), the high light harvesting efficiency (LHE, 0.9984) and the smallest internal reorganization energy (*λ*_int_, 5.05 kcal mol^−1^). Hence, A6 not only enhances electronic excitation, but also improves the reorganization energy. Importantly, A6 shows the largest red shift and the maximum integral values of the adsorption over the visible light, as well as the strongest adsorption energy (−74.80 kcal mol^−1^) on a NiO surface. Thus, A6 may be a promising sensitizer for the p-type dye-sensitized solar cells (DSSCs), and the acceptor of A6 may provide a new and easily accessible high performance acceptor for p-type sensitizers.

## Abstract 2

Based on triphenylamine as an electron donor and thiophene as a π-linker, Series P and A p-type sensitizers were designed to investigate the effects of the different acceptors on the properties of the sensitizers.

## Graphical abstract

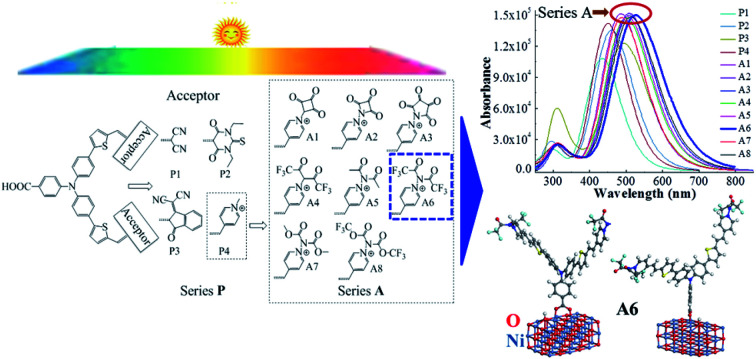

## 1. Introduction

Dye-sensitized solar cells (DSSCs) attracted great interest after O'Regan and Grätzel's report about the sensitization of an n-type semiconductor TiO_2_,^1^ due to the potential of being environment-friendly and low-cost. Most of the fundamental and applied studies have been conducted on n-type DSSCs based on TiO_2_ semiconductors, which have a solar energy conversion efficiency (*η*) of 14.3%.^2^ However, the light harvesting efficiency of the p-type DSSCs is as low as 2.51%.^3^ Therefore, the development of pn-type DSSCs is limited by the low efficiency of p-type DSSCs. More researches on p-type sensitizers are needed for future applications.

Typical sensitizers for DSSCs are based on a donor–spacer–acceptor system (D–π–A), to achieve effective charge separation and transfer.^4^ Based on p-type sensitizers with the D–π–A structure, the π-linker and the donor are often modified, or new groups are added between donor and acceptor to form a D–D–π–A, D–A–π–A or D–π–π–A structure in order to improve the performance of dyes.^5–7^ However, there are few studies on the modification of acceptors with the introduction of electron withdrawing groups, which can promote the photoelectron transition towards the acceptor. Dicyanoethene as an acceptor of the dye is widely used in p-type DSSCs, and many different acceptors also have been synthesized and used in p-type sensitizers.^7–9^ Pyridinium derivatives have mature synthetic routes and wide applications.^10^ Recently, p-type sensitizers based on *N*-methylpyridinium as the acceptor have been synthesized and *N*-methylpyridinium has been proved to be a good acceptor.^11^ The electron withdrawing groups have a significant impact on the performance of dyes, thus introducing the electron withdrawing group in pyridinium acceptor may improve the properties of dyes.

**Fig. 1:**
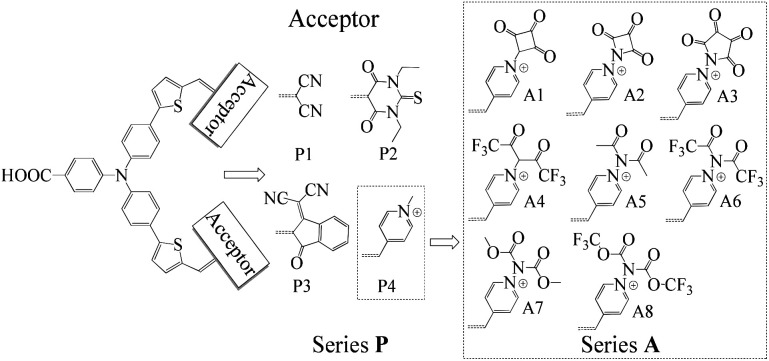
Molecular structures of the investigated dyes.

The synthesis of new sensitizers is time consuming, hence, theoretical study is considered as an efficient way to investigate the relationship between the molecular structures and the chemical properties of dyes. P1 dye with triphenylamine as an electron donor, thiophene as π-linker and dicyanoethene as an acceptor has been synthesized and proved to be a good p-type sensitizer.^12^ In this study, based on triphenylamine as an electron donor and thiophene as π-linker, 1,3-diethyl-2-thiobarbituric acid, 3-dicyanovinylindan-1-one and *N*-methylpyridinium that have been synthesized and used in p-type DSSCs were employed to replace the malonitrile acceptor of P1, which together with P1 form Series P dyes. Furthermore, on the basis of a prototype P4 with *N*-methylpyridinium as acceptor, eight kinds of electron withdrawing groups were introduced in the N atom of pyridinium as new acceptors. These eight kinds of dyes form Series A dyes. The properties of Series A dyes and Series P dyes are calculated by the density functional theory (DFT) and time-dependent DFT (TD-DFT). The molecular structures of all investigated dyes were shown in Fig. 1. By comparing the molecular structures and the properties of all dyes, we revealed how the different electron-withdrawing groups affect the properties of dyes. We expected that the results would boost the development of p-type photosensitizers.

## 2. Computational methods and modelling

The molecular structure and electronic properties for all investigated dyes were calculated by the quantum chemical program Gaussian 09.^13^ The equilibrium geometry of the dyes in neutral, cationic and anionic states were optimized at the B3LYP/6-311G** level, which was widely used for p-type triphenylamine system.^14,15^ The excited state properties were calculated at CAM-B3LYP/6-311G** level by the TD-DFT methods, which was widely used to predict the properties of excited states.^16,17^ The whole Gaussian calculations were performed under acetonitrile as the solvent and the polarized continuum model (PCM) throughout. The structures of the dye/NiO system were optimized at the GGA-PBE/DN level under acetonitrile as the solvent, using the Dmol^3^ program of Materials Studio 6.0.^18,19^ The adsorption energies between the dye and the NiO surface were calculated at the same level.

## 3. Results and discussion

### 3.1 Electronic properties of dyes

**Fig. 2:**
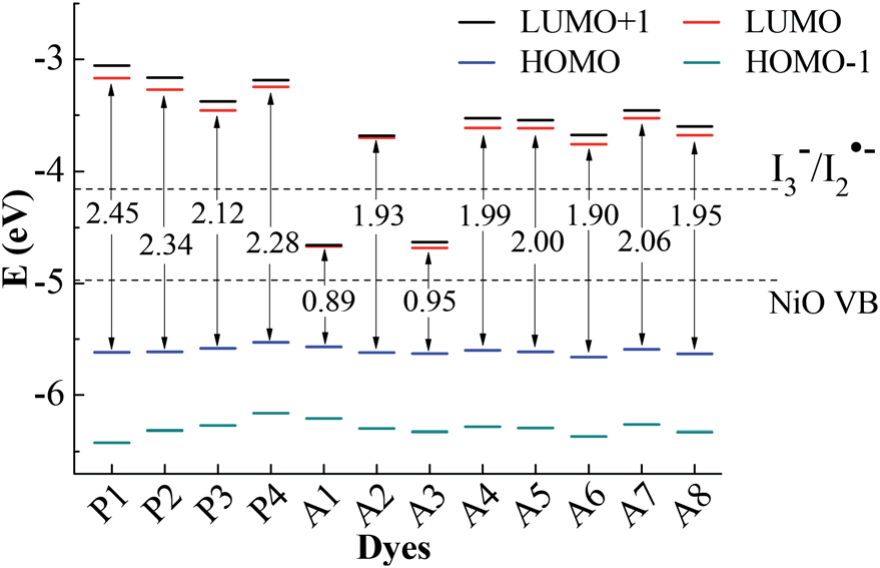
Frontier molecular orbital energy levels and energy gap, together with *E*_VB_ (NiO) and *E*(*I*_3_^−^/*I*_2_˙^−^).

For the p-type DSSCs, the HOMO potential levels must be lower than the NiO valence band, while the LUMO potential levels must be higher than *I*_3_^−^/*I*_2_˙^−^ redox potential,^20,21^ which can ensure an efficient and fast hole transfer and separation. Fig. 2 shows the frontier molecular orbital energy levels calculated at the B3LYP/6-311G** level for all dyes, as well as the valence band levels of semiconductor NiO (*E*_VB_, −4.98 eV) and the redox potential of the mediator *E*(*I*_3_^−^/*I*_2_˙^−^, −4.15 eV).^22,23^ Although the redox potential of the *I*_3_^−^/*I*^−^ redox couple is often selected as the reference potential of p-type DSSCs, it is proved by experiments that there are two reaction steps (*I*_3_^−^ + e^−^ → *I*_2_˙^−^ + *I*^−^ and *I*_2_˙^−^ + e^−^ → 2*I*^−^) in the regeneration process of *I*_3_^−^/*I*^−^.^24^ The redox potential of *I*_3_^−^/*I*_2_˙^−^ is higher than that of *I*_3_^−^/*I*^−^. Only when the LUMO level of the dye is higher than the redox potential of *I*_3_^−^/*I*_2_˙^−^, the dye regeneration can proceed spontaneously. In Gibson's research, the redox potential of *I*_3_^−^/*I*_2_˙^−^ in acetonitrile is −4.28 eV. In theoretical studies, the redox potential of *I*_3_^−^/*I*_2_˙^−^ is usually considered as −4.15 eV. Therefore, it is more accurate to judge whether the dye is the p-type dye by comparing with the value of the redox potential of *I*_3_^−^/*I*_2_˙^−^. As can be seen in Fig. 2, the LUMO levels for all dyes except A1 and A3 are above the *I*_3_^−^/*I*_2_˙^−^ redox couple and the HOMO levels are all below NiO valence band (VB). This implies that A1 and A3 are unsuitable to be used as sensitizers for the p-type DSSCs. All the other ten dyes match well with the NiO semiconductor electrode and *I*_3_^−^/*I*_2_˙^−^ electrolyte, and can be used as p-type sensitizers. The frontier molecular orbitals are closely related to the properties of the dyes, especially the HOMO and LUMO. Therefore, the study of the influence of the different structural acceptors of dyes on the HOMO and LUMO levels is necessary.

As shown in Fig. 2, the HOMO levels of all the studied dyes vary very little, while the LUMO levels change a lot. This is due to the fact that all the investigated dyes have the same donor and π-linker groups but different acceptors. Compared with P1, the LUMO levels of the other dyes move towards a more negative potential, especially for Series A dyes. Generally, the stronger the electron-withdrawing ability of the acceptor, the easier the intramolecular charge transfer towards acceptor,^25^ which will cause the LUMO level of the dye to move towards a more negative potential.^26^ When the electron-withdrawing group is attached to the acceptor of P4, the LUMO level of the modified dye moves down. Thus, the LUMO levels of Series A dyes are lower than P4. For A1, the strong electron-withdrawing ability of 1,2,3-cyclobutanone in the acceptor results in the LUMO level falling below the *I*_3_^−^/*I*_2_˙^−^ redox potential. The structural difference between A1 and A2 is only the N^+^–C bond and the N^+^–N bond in the acceptors. For A2, the lone pair electrons on the N atom in the quaternion ring of the acceptor can form a conjugated ring with the three C atom connecting ketone group in the quaternion ring. The conjugated quaternion ring in the acceptor of A2 can significantly weaken the electron-withdrawing ability of the acceptor. Therefore, the LUMO level of A2 is much higher than A1. The acceptor of A3 also contains a conjugated ring, but the LUMO level is below the *I*_3_^−^/*I*_2_˙^−^ redox potential. Compared with A2, A3 has more ketone groups in its acceptor, so A3 has a stronger electron-withdrawing ability than A2. This leads to the LUMO level of A3 much lower than those of A2 and the *I*_3_^−^/*I*_2_˙^−^ redox couple. The difference between the A4 and A6 is also only the N^+^–C bond and the N^+^–N bond in the acceptors. The lone pair electrons on the N atom in the acceptor of A6 can form a conjugated system with the two adjacent ketone groups, which makes the intramolecular charge transfer towards acceptor more easily, but the C atom in the acceptor of A4 cannot conjugate with the ketones. Thus, the LUMO level of A6 moves towards a more negative potential slightly in comparison with A4. For A5, the electron-withdrawing ability of methyl is weaker than trifluoromethyl in the acceptor of A6, so the LUMO level of A5 moves down in comparison with that of A6. As can be seen in Fig. 2, the LUMO level of A7 and A8 is higher than that of A5 and A6, respectively. This is because that the electron-withdrawing ability of the ester group (–COOR) is weaker than the acyl group (–COR). For p-type DSSCs, one of the most important factors affecting dye performance is the efficient hole injection.^27^ The low HOMO level is very important for p-type sensitizers. As shown in Fig. 2, the HOMO levels of Series A dyes are similar with P1, so the Series A dyes maintain the hole injection ability as P1. A6 has the lowest HOMO level in all the dyes. This is beneficial to the hole injection from the excited dye to the semiconductor.

The HOMO–LUMO energy gaps of all the dyes are also shown in Fig. 2. The energy gaps of Series A dyes are smaller than those of Series P. For DSSCs sensitizers, smaller gaps requires less energy for the electron transitions, which is beneficial for absorbing light at longer wavelengths.^28^ This guarantees a larger light harvesting and a prerequisite for overall power conversion efficiency of sunlight.^29^ Thus, the introduction of the electron-withdrawing groups to the N atom in pyridinium acceptor is an effective way to improve the electronic properties, and also can regulate the energy levels and the energy gap of dyes. The energy gap of A6 is only 1.90 eV, which is the narrowest of all the dyes except for A1 and A3 that cannot be used as p-type sensitizers. Therefore, the introduction of the acceptor of A6 may improve the performance of the dye and A6 may be a promising dye for the p-type DSSCs.

In order to obtain a good electron “push–pull” effect, to ensure that the photo-excited electron transfer from the donor to acceptor and to reduce the combination of electron and hole, the HOMO distribution of p-type dyes should be significantly near the anchor side, and the LUMO distribution of dyes should locate far away from the NiO to reduce the hole combination.^28^ The contours of molecular frontier orbitals for the ten p-type dyes are displayed in Table 1. As can be seen in Table 1, the HOMOs distribute mainly on the “D–π–” groups in “D–π–A” system, while LUMOs dominantly on the “–π–A” groups. For the ten p-type dyes, the electrons can be delivered from the donors to the acceptors smoothly.

**Table 1:** Contours of molecular frontier orbitals of dyes

Dyes	HOMO	LUMO	Dyes	HOMO	LUMO
P1	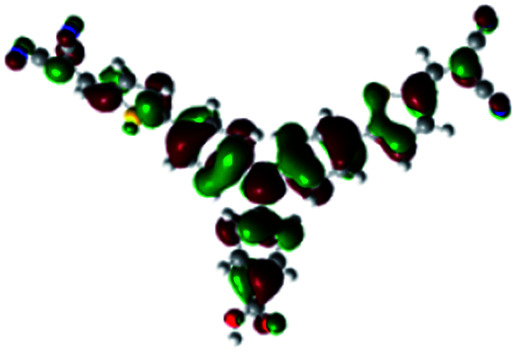	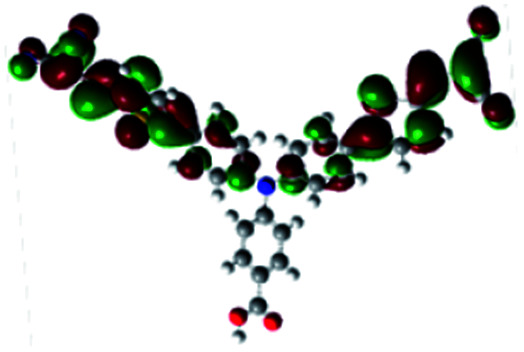	P2	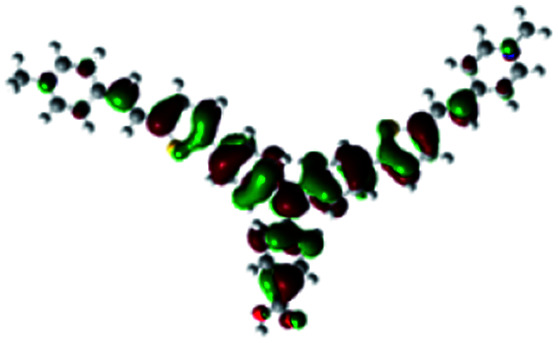	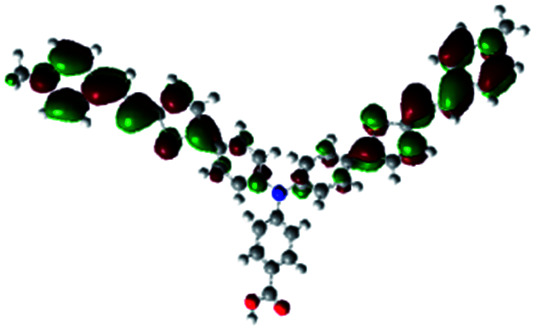
P3	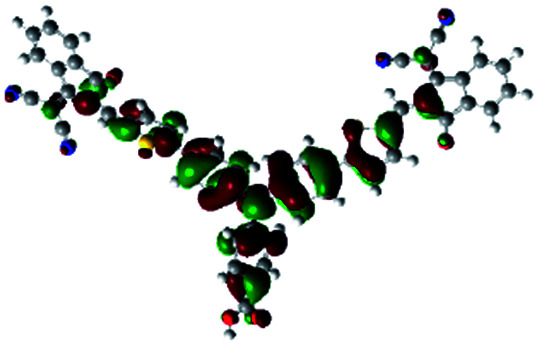	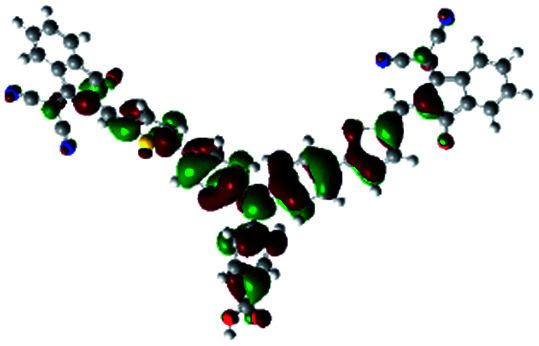	A0	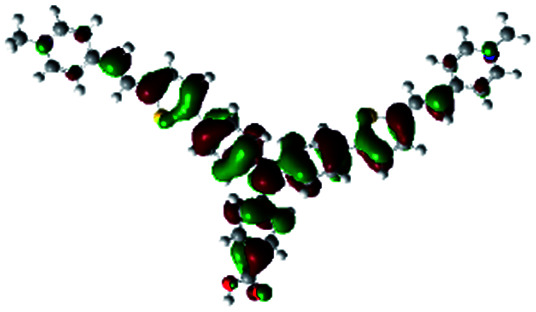	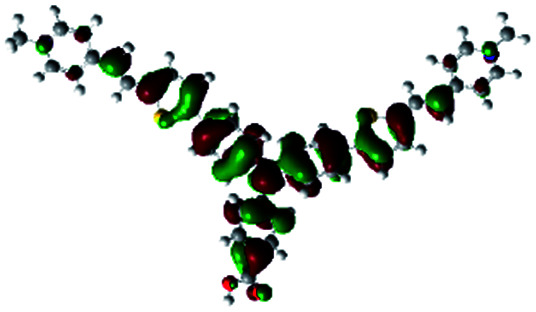
A2	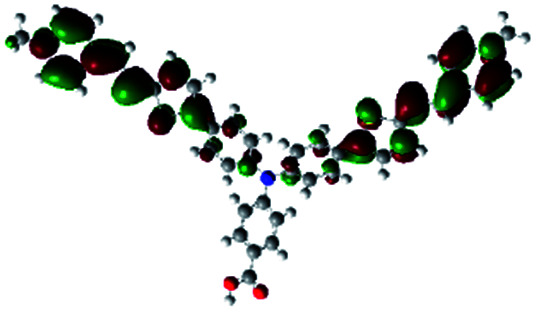	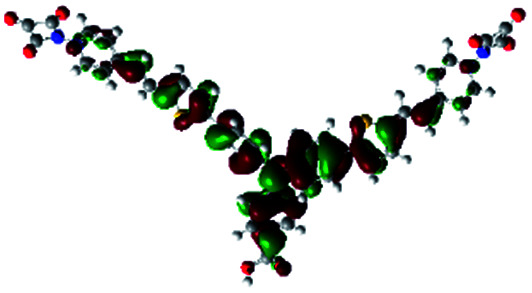	A4	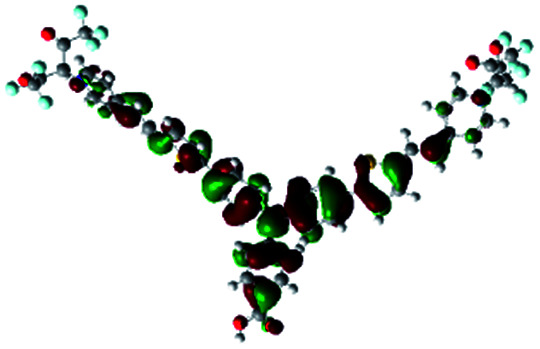	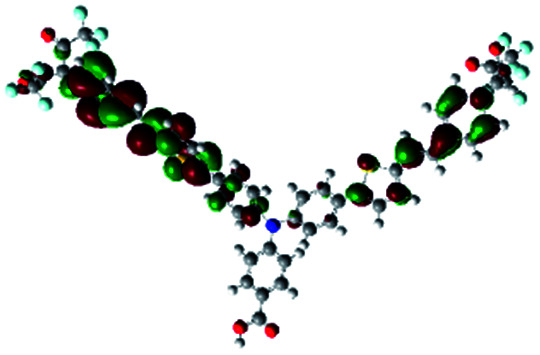
A5	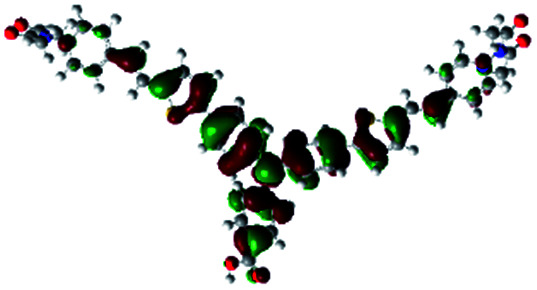	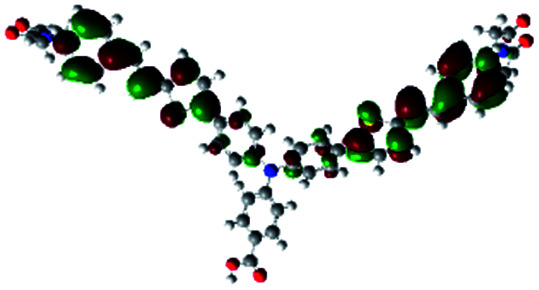	A6	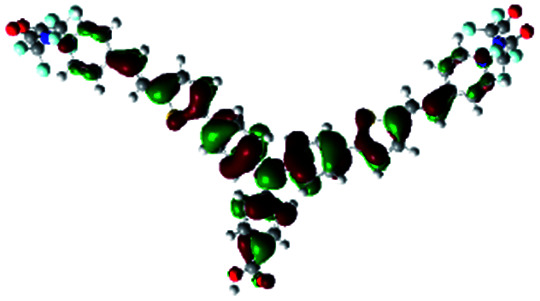	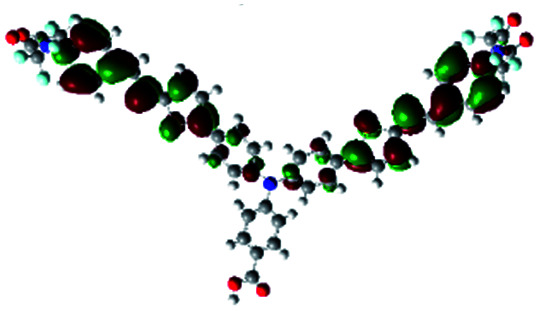
A7	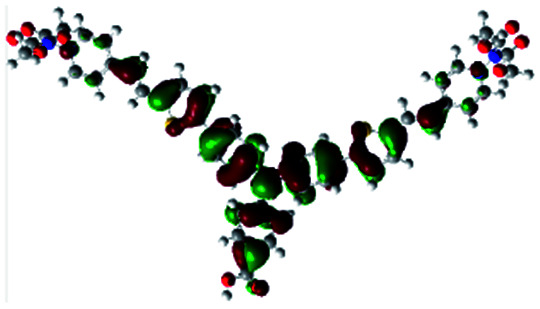	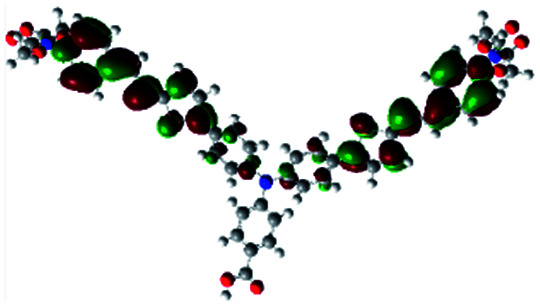	A8	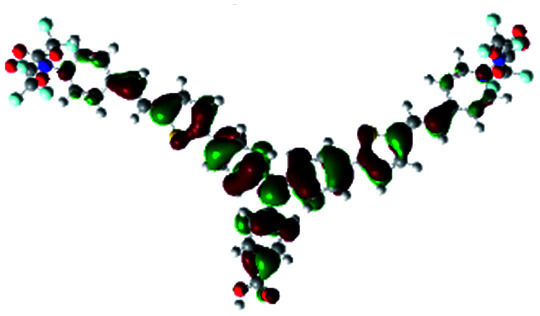	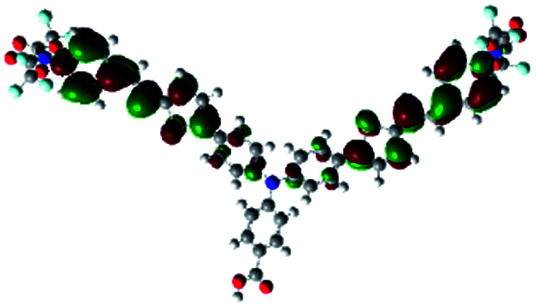

### 3.2 UV/vis absorption spectra

UV-vis absorption spectrum is an important characteristic to evaluate the photovoltaic properties of dyes for DSSCs. In order to obtain the reliability of the calculation, the UV-vis absorption spectrum of P1 was simulated *via* five functionals of B3LYP, LC-BLYP, WB97XD, CAM-B3LYP and PBE1PBE with 6-311G** basis set. A comparison of the simulated UV-vis absorption spectra of P1 with its experimental spectrum is made and displayed in Fig. S1.† The shape of the absorption-wavelength curve for P1 calculated by CAM-B3LYP functional is the most similar to its experimental spectrum. For P1, the maximum absorption wavelength of the simulated spectrum at the CAM-B3LYP/6-311G** level and the experimental spectrum is 434 nm and 468 nm, respectively.^12^ The deviation of the two maximum absorption wavelengths is 34 nm. Therefore, the simulated UV-vis absorption spectra for all the investigated dyes were calculated at the CAM-B3LYP/6-311G ** level.

**Fig. 3:**
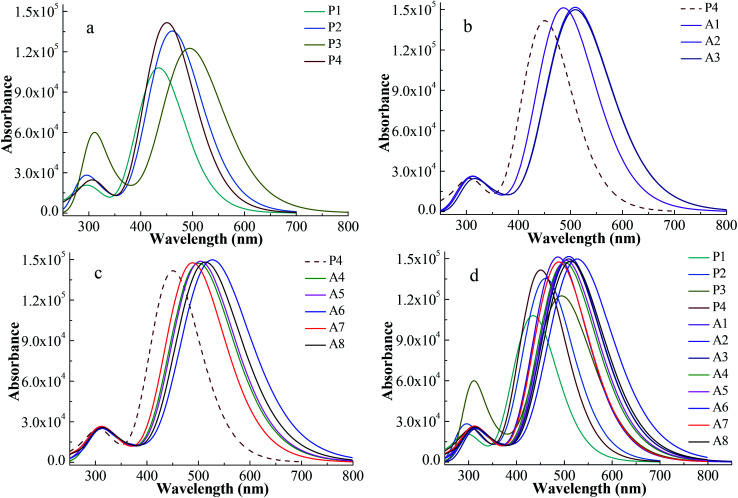
Absorption spectra for Series P dyes (a), Series A with cyclic ketone groups (b), Series A with ester or aliphatic ketone groups (c) and overall dyes (d) at CAM-B3LYP/6-311G** level.

The simulated UV-vis absorption spectra were displayed in Fig. 3 and their simulated values were list in Table 2. A high efficiency dye should have a strong and broad absorption over the visible light (400–800 nm). The boundary of the absorption peaks is hard to define. Hence, the full width at half maximum (FWHM) of the strongest absorption peak can be used to describe the width of the absorption peak.^30^Fig. 3a shows the spectra of Series P dyes. The order of the maximum absorption wavelength (*λ*_max_) for Series P is P3 > P2 > P4 > P1, which is consistent with the order of the energy gaps of them (Fig. 2). In general, the narrower the energy gap, the more the red shift of UV-vis absorption.^31^P4 has the strongest the intensity at the maximum absorption peak in Series P, which is beneficial for absorbing more photons. But P4 has no advantage in the UV-vis absorption of visible light because it displays a blue shift of the absorption over 400–800 nm in comparison with P2 and P3. The electron-withdrawing group of P4 is N^+^. The introduction of the electron-withdrawing groups on the N^+^ can improve the electron-withdrawing ability of the acceptor. This will facilitate electron transfer from the donor to the anchor, thereby causing a red shift of the absorption for P4. As shown in Fig. 3b, the dyes of A1, A2 and A3 show red shifts and UV-vis absorption enhancement in comparison with P4 due to the introduction of the electron-withdrawing cyclic ketone on the N^+^. The UV-vis absorption spectrum for A2 shows a red shift in comparison with A1, while the difference of the UV-vis absorption between A2 and A3 is very small. The maximum absorption peak of the UV-vis spectrum mainly originates from the main configurations of the electron transitions, and the detail discussion will be followed in Section 3.3 and Table 3. Fig. 3c displays the spectra of the A4–A8 dyes, which also show red shifts and UV-vis absorption enhancement in comparison with P4. As shown in Table 2, the order of the *λ*_max_ for A4–A8 is A6 > A8 > A5 > A4 > A7, which is consistent with the order of the energy gaps of them (Fig. 2). For A4–A8 dyes, the electron withdrawing ability of the acceptors determines the levels of HOMO and LUMO, as well as the shifts of their spectra. A6 has the largest red shift and the strongest and broadest absorption over the visible light, due to the introduction of the hexafluorodiacetylamino group on N^+^. Fig. 3d shows the spectra of all the studied dyes. There are two main absorption peaks for all the dyes, which are attributed by the intramolecular charge transfer of π–π* electron transition.^32^ As can be seen in Fig. 3d, Series A dyes are more beneficial to the absorption of visible light in comparison with Series P. A6 has the similar intensity of the maximum absorption peak to the other Series A dyes, but it has the largest red shift (*λ*_max_ = 531.0 nm) and the broadest absorption (FWHM = 162.7 nm) over the visible light.

**Table 2:** The maximum absorption wavelength (*λ*_max_), the full width at half maximum of the strongest absorption peak

Dyes	*λ*_max_ (nm)	FWHM (nm)	Dyes	*λ*_max_ (nm)	FWHM (nm)
P1	434	110.1	P2	460.6	123.7
P3	494.4	141.8	P4	450.8	115.1
A1	477.6	129.3	A2	510.4	147.9
A3	511.9	148.7	A4	502.4	145.3
A5	505.6	146.2	A6	531.0	162.7
A7	491.2	137.8	A8	515.2	152.7

**Table 3:** Computed Δ*G*_inj_, Δ*G*_reg_, Δ*G*_CR_, transition configuration and LHE of the dyes

Dyes	Δ*G*_inj_ (eV)^a^	Δ*G*_reg_ (eV)^b^	Δ*G*_CR_ (eV)^c^	*f*	Main configurations	LHE
P1	−0.64	−0.98	−1.81	1.9641	H → L (73%), H−1 → L+1 (18%)	0.9891
P2	−0.63	−0.88	−1.71	2.5576	H → L (68%), H−1 → L+1 (20%)	0.9972
P3	−0.60	−0.69	−1.52	2.1978	H → L (62%), H−1 → L+1 (23%)	0.9937
P4	−0.55	−0.90	−1.73	2.5836	H → L (60%), H−1 → L+1 (26%)	0.9974
A1	−0.59	0.52	−0.31	2.6678	H → L+2 (60%), H−1 → L+3 (25%)	0.9979
A2	−0.64	−0.46	−1.29	2.8133	H → L (63%), H−1 → L+1 (24%)	0.9985
A3	−0.65	0.53	−0.30	2.8433	H → L+2 (63%), H−1 → L+3 (24%)	0.9986
A4	−0.62	−0.54	−1.37	2.5435	H → L (59%), H−1 → L+1 (17%)	0.9971
A5	−0.63	−0.53	−1.36	2.7948	H → L (63%), H−1 → L+1 (24%)	0.9984
A6	−0.68	−0.39	−1.22	2.8010	H → L (65%), H−1 → L+1 (23%)	0.9984
A7	−0.61	−0.62	−1.45	2.7106	H → L (62%), H−1 → L+1 (25%)	0.9981
A8	−0.65	−0.47	−1.30	2.8193	H → L (64%), H−1 → L+1 (23%)	0.9985

a Δ*G*_inj_ = *E*_HOMO_ − *E*_VB_(NiO).

b Δ*G*_reg_ = *E*(*I*_3_^−^/*I*_2_˙^−^) − *E*_LUMO_.

c Δ*G*_CR_ = *E*_LUMO_ − *E*_VB_(NiO).^36,37^

In order to reduce the deviation between the theoretical and experimental value, the diffuse basis set of 6-311++G** was chosen to simulate the UV-vis absorption spectra of P1 and some selected dyes. The simulated UV-vis absorption spectra at the CAM-B3LYP/6-311++G** level were shown in Fig. S2.† The results show that the *λ*_max_ of the simulated spectrum for P1 at CAM-B3LYP/6-311++G** level is 441 nm, which reduces the deviation from the experimental value of 468 nm in comparison with 434 nm calculated at CAM-B3LYP/6-311G** level. Compared with the results at CAM-B3LYP/6-311G** level, the spectrum displays a red shift when the diffuse basis set is added. But the relative positions of the *λ*_max_ for the dyes hardly change. Importantly, the conclusions of the two calculation levels are consistent. Therefore, the conclusions obtained from Fig. 3 are reliable.

**Fig. 4:**
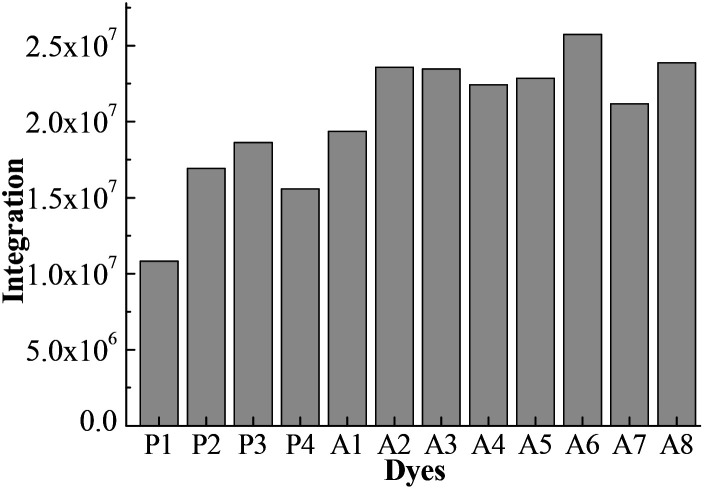
The integral area of absorption–wavelength curve over 400–800 nm.

Fig. 4 shows the integral area of the absorption–wavelength curve for all dyes over 400–800 nm. The proportion of ultraviolet light in sunlight is very small. The absorption for a good solar sensitizer should show a slightly red shift in comparison with the visible light. Therefore, we chose 400–800 nm to calculate the integral area for all dyes, which can evaluate the photovoltaic properties of dyes for DSSCs. The integral values of Series A dyes are larger than those of Series P. The introduction of the electron-withdrawing groups to the N^+^ in the acceptor of P4 is an effective way to improve the absorption of visible light and the photovoltaic properties of dyes. A6 has the largest the integral area of the absorption–wavelength curve over 400–800 nm. Therefore, for p-type DSSCs, A6 may be a promising dye, and the dyes with the acceptor of A6 may have the good photovoltaic properties of DSSCs.

### 3.3 Performances of p-type dyes

The energy conversion efficiency (*η*) is proportional to both the open-circuit photovoltage (*V*_oc_) and the short-circuit photocurrent density (*J*_sc_).^26^ Under the same electrode conditions, the *J*_sc_ is determined by the light-harvesting efficiency (LHE) and the driving forces of hole injection (Δ*G*_inj_) from the excited dye to the semiconductor, dye regeneration (Δ*G*_reg_) between the oxidized dye and the electrolyte, and charge recombination (Δ*G*_CR_) from the oxidized dye to the semiconductor.^33^ The more negative Δ*G*_inj_ and Δ*G*_reg_ are beneficial for hole injection and dye regeneration.^34^ However, the more negative the Δ*G*_CR_ is, the more likely the charge recombination will occur to some extent.^34^

The computed Δ*G*_inj_, Δ*G*_reg_, Δ*G*_CR_, transition configuration and LHE of the dyes are displayed in Table 3. As can be seen in Table 3, the Δ*G*_reg_ for A1 and A3 is positive, which indicates that the dyes A1 and A3 cannot be effectively regenerated. This is consistent with the conclusion that A1 and A3 are not suitable to be used as p-type sensitizers mentioned above. For p-type DSSCs dyes, the electron-withdrawing anchor is located in the donor units, thus one of the important factors affecting the properties of dyes is the efficient hole injection, thereby Δ*G*_inj_ is more important for p-type dyes.^27^ The Δ*G*_inj_ for all dyes is in the range of −0.68 to −0.55 eV. The Δ*G*_inj_ of A6 is −0.68 eV, which is the best in all dyes. In addition, the Δ*G*_CR_ values for all dyes except for A1 and A3 are in the range of −1.81 to −1.22 eV. The Δ*G*_CR_ of A6 is −1.22 eV, indicating the smallest driving force of charge recombination in all dyes. Typically, efficient dye regeneration can occur when the driving force is larger than 0.2 eV.^35^ The Δ*G*_reg_ of A6 is −0.39 eV. The value of the Δ*G*_reg_ can ensure that dye regeneration of A6 will be smoothly processed. Therefore, compared with the other dyes, A6 may effectively improve the hole injection and suppress the charge recombination to some extent as a p-type dye.

According to the transition configurations shown in Table 3, the HOMO to LUMO transition is the largest portion of transition configuration except A1 and A3 that cannot be used as p-type dyes, and the HOMO−1 and LUMO+1 also participate in the transition. For A1 and A3, the largest portion of transition configuration is HOMO to LUMO+2, and the second largest portion of transition configuration is HOMO−1 and LUMO+3. The maximum absorption peak of the UV-vis spectrum mainly originates from the main configurations of the electron transitions.^38^ The larger the proportion of HOMO and LUMO participating in the transition, the more favorable the hole injection from the dye to the valence band of semiconductor NiO.^39^ The three largest contributions of HOMO to LUMO transition for all dyes are belonged to P1, P2 and A6. The contribution of HOMO to LUMO transition of A6 is 65%, which is the largest in Series A.

The LHE is closely related to the oscillator strength (*f*) of dye and can be approximated as:^23^

1 LHE ≈ 1 − 10^−*f*^

As can be seen in Table 3, compared with the other dyes of Series P, the LHE value for P4 is the largest. For Series A dyes, the values of LHE are further increased except for A4. This indicates that the introduction of the electron-withdrawing groups in the acceptor of P4 is beneficial to improve LHE of dyes. A2, A3, A6 and A8 have the largest four LHE values but A3 cannot be used as p-type dye. The LHE values for A2, A8 and A6 are 0.9985, 0.9985 and 0.9984, respectively.

The low efficient hole injection and the fast charge recombination limit the performance and development of the p-type DSSC.^27^ Therefore, the reorganization energy for all dyes was calculated. The reorganization energy consists of the external (*λ*_ext_) and internal (*λ*_int_) reorganization energies.^7,27^*λ*_ext_ is often ignored because the solvent has little effect on the charge-transfer dynamics when the solvent is same. Hence, *λ*_int_ is the main influence on the total reorganization energy, which can be approximately calculated as the following formula:^7^

2 *λ*_int_ = *λ*_+_ + *λ*_−_

3
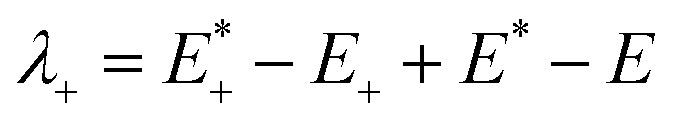
4
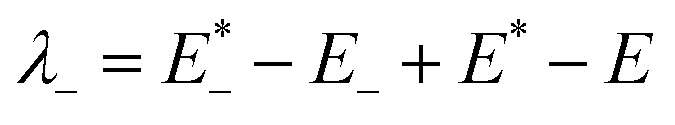
where *λ*_+_ is the hole reorganization energy, *λ*_−_ is the electron reorganization energy, 
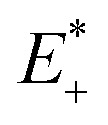
 is the single point energy of dye cation at the neutral geometry, *E*_+_ is the optimized energy of the cationic dye, E* is the single point energy of the neutral dye at its cation geometry, *E* is the optimized energy of the neutral dye, 
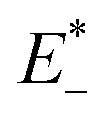
 and *E*_−_ are the corresponding energies for anion.

On the basis of the Marcus theory, *λ*_+_ can affect the kinetics of hole injection, and *λ*_−_ can affect the kinetics of electron injection.^40^ The smaller *λ*_+_ or *λ*_−_ can result in a higher yield of hole or electron injection.^22^ The calculated reorganization energies for all dyes were list in Table 4. The values of *λ*_int_ for A1, A2 and A3 are obviously larger than the other investigated dyes. This implies that the conversion between neutral molecule and its anion is difficulty to occur. The *λ*_int_ of A6 is 5.05 kcal mol^−1^, which is the lowest among all the dyes. The low *λ*_int_ of A6 will be beneficial to the hole injection. Therefore, A6 may be a promising dye that increases the efficient hole injection of dyes, and the dyes with the acceptor of A6 may have good properties as p-type dyes.

**Table 4:** Reorganization energies calculated at the B3LYP/6-311G** level

Dyes	*λ*_+_ (kcal mol^−1^)	*λ*_−_ (kcal mol^−1^)	*λ*_lint_ (kcal mol^−1^)
P1	2.70	5.85	8.55
P2	2.91	6.37	9.29
P3	2.84	2.62	5.47
P4	3.97	5.72	9.69
A1	3.46	16.82	20.28
A2	3.22	15.27	18.49
A3	3.15	13.56	16.71
A4	3.14	4.56	7.69
A5	3.20	2.26	5.46
A6	2.94	2.12	5.05
A7	3.45	2.58	6.03
A8	3.08	2.39	5.47

### 3.4 The dye/NiO interaction

In DSSCs, the interaction between dye and semiconductor interface has a significant impact on hole injection efficiency.^41^ In general, the value of the adsorption energy for the dye/NiO system determines the strength of the interaction between the dye and the surface of NiO semiconductor.^19^ A large adsorption energy can lead to a strong electronic coupling between the anchoring unit of the dye and the NiO surface, which can also increase the hole transfer rate.^41^

**Fig. 5:**
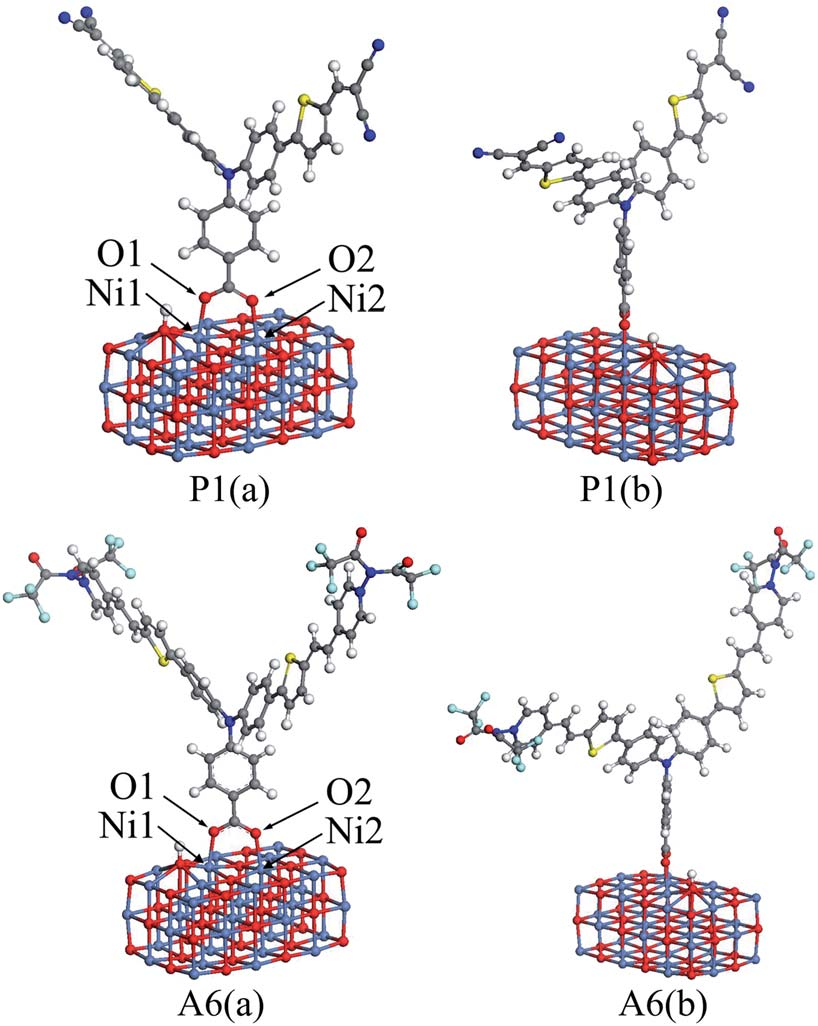
The front view (a) and lateral view (b) of the optimized dye/(NiO)_12×3_ structures for P1 and A6.

To calculate the adsorption energies, a nickel oxide cluster with three layers of 12 × 3 NiO were established, and the (NiO)_12×3_ cluster was optimized at the GGA-PBE/DN level under a acetonitrile solvation model, using the Dmol^3^ program. For all dyes, the optimized structure was located on the optimized (NiO)_12×3_ cluster, respectively. The bottom two NiO layers for all the dye/NiO systems were fixed, while other atoms were allowed to relax. Each dye/NiO system was optimized at the same level as the (NiO)_12×3_ cluster. The optimized configurations for P1/(NiO)_12×3_ and A6/(NiO)_12×3_ were presented as examples in Fig. 5.

The dyes bind almost perpendicular onto the NiO surface through the bidentate coordination bridging with the formation of two interface O–Ni bonds. The bridging bidentate coordination mode is more preferable and stable than the other binding modes, thereby adding the rate of hole injection.^42,43^ The calculated bond lengths of Ni1–O1 and Ni2–O2 and the adsorption energies are displayed in Table 5 for all dyes. The Ni–O bond lengths are in the range of 2.01 to 2.05 Å, which are in good agreement with the value from the literature.^44^ This indicates that the calculation level chosen is suitable. As seen in Table 5, the adsorption energies for Series A are larger than that of P4. This reveals that the introduction of the electron-withdrawing groups to the N^+^ will enhance the interaction between dyes and NiO electrode. The adsorption energy of P2 is −43.87 kcal mol^−1^, which is the lowest in all dyes. The adsorption energy of A6 is −74.80 kcal mol^−1^, which is the highest except for A3 that is unsuitable as p-type sensitizers. The acceptors of Series P dyes have been synthesized and used as p-type sensitizers in many studies.^1,8,9,11^ Compared with Series P, the large adsorption energy of A6 may lead to an increase in the hole transfer rate between dye and semiconductor interface. This is very important for improving the properties of p-type sensitizers.

**Table 5:** Adsorption energy and bond lengths between dye and (NiO)_10×3_

Dyes	Adsorption energy kcal mol^−1^	Band length (Å)	
		Ni1–O1	Ni2–O2
P1	−44.06	2.02	2.01
P2	−43.87	2.02	2.04
P3	−54.32	2.02	2.02
P4	−46.47	2.02	2.02
A1	−67.92	2.03	2.03
A2	−69.29	2.03	2.04
A3	−75.93	2.02	2.02
A4	−72.99	2.02	2.02
A5	−58.91	2.03	2.05
A6	−74.80	2.03	2.04
A7	−54.00	2.02	2.02
A8	−64.21	2.02	2.02

## 4. Conclusions

On the basis of DFT and TD-DFT, Series P and A p-type sensitizers with D–(A–π–A)_2_ structure were designed to investigate the effect of the different acceptors on the properties of the dyes. The results show that the properties of the dyes could be tuned by the introduction of the electron-withdrawing groups to the N atom in pyridinium acceptor. By comparing the properties of all the other dyes, A6 has the narrowest energy gap, the largest driving force of hole injection and the smallest internal reorganization energy. Both of the electronic excitation and the reorganization energy of A6 are improved in comparison with the other dyes. A6 not only shows the largest red shift of the UV-vis absorption and the maximum integral values of the adsorption over visible light, but also displays the strongest adsorption energy on NiO surface. Therefore, A6 may be a promising dye for the p-type DSSCs and its acceptor is an easily accessible new acceptor for p-type sensitizers.

## Conflicts of interest

There are no conflicts to declare.

## Supplementary Material

RA-010-D0RA00610F-s001
