# Supplementary material for: Design of high performance p-type sensitizers with pyridinium derivatives as the acceptor by theoretical calculations
Source: RSC Adv. 2020 Mar 12;10(18):10569–76. doi: 10.1039/d0ra00610f (PMC9050369; doi:10.1039/d0ra00610f)
Supplement: RA-010-D0RA00610F-s001 [file RA-010-D0RA00610F-s001.pdf]

Electronic Supplementary Material (ESI) for RSC Advances.
This journal is © The Royal Society of Chemistry 2020

Supporting information

Design of high performance *p*-type sensitizers with pyridinium derivatives as acceptor by theoretical calculations

Zhi-Dan Sun,[a] Jiang-Shan Zhao,[a] Karuppasamy Ayyanar,[a] Xue-Hai Ju[a*] and Qi-Ying Xia[b*]

[a] *Key Laboratory of Soft Chemistry and Functional Materials of MOE, School of Chemical Engineering, Nanjing University of Science and Technology, Nanjing 210094, P. R. China. Email: xhju@njust.edu.cn*

[b] *School of Chemistry and Chemical Engineering, Linyi University, Linyi, P. R. China.*

Email: xiaqiying@163.com

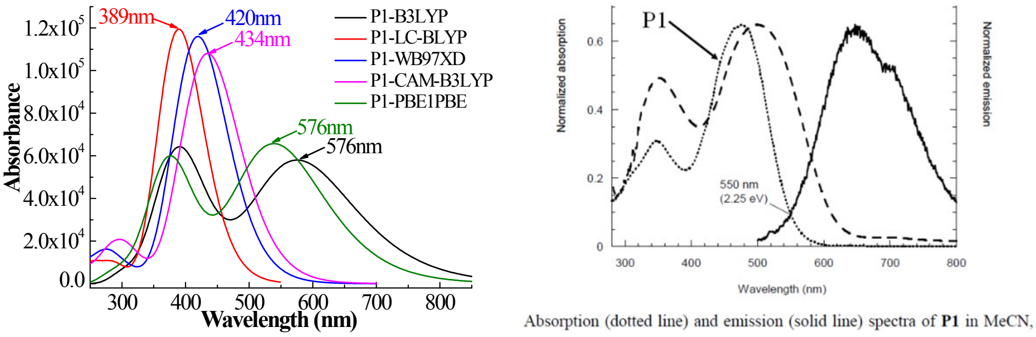

Absorption (dotted line) and emission (solid line) spectra of **P1** in MeCN,

Fig. S1 The simulated UV-vis absorption spectrum of **P1** by different functionals using the same basis set 6-311G**, as well as its experimental spectrum

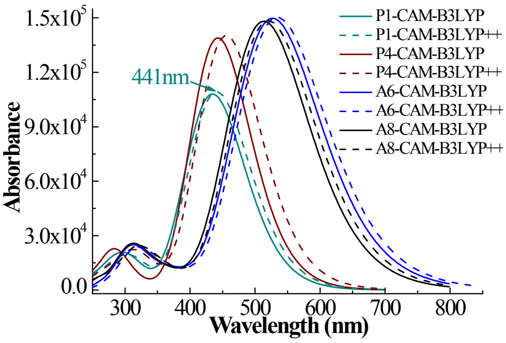

Fig. S2 The simulated UV–vis absorption spectra for some selected dyes at CAM-B3LYP/6-311++G** level
